# Molecular diagnostics helps to identify distinct subgroups of spinal astrocytomas

**DOI:** 10.1186/s40478-021-01222-6

**Published:** 2021-06-30

**Authors:** Annamaria Biczok, Felix L. Strübing, Julia M. Eder, Rupert Egensperger, Oliver Schnell, Stefan Zausinger, Julia E. Neumann, Jochen Herms, Joerg-Christian Tonn, Mario M. Dorostkar

**Affiliations:** 1 Department of Neurosurgery, Ludwig-Maximilians-University Munich, Marchioninistr. 15, 81377 Munich, Germany; 2 Partner Site Munich, and German Cancer Research Center (DKFZ), German Cancer Consortium (DKTK), Heidelberg, Germany; 3 Center for Neuropathology and Prion Research, Ludwig-Maximilians-University Munich, Munich, Germany; 4 Department of Neurosurgery, Medical Center – University of Freiburg, Freiburg, Germany; 5 Institute of Neuropathology, University Medical Center Hamburg-Eppendorf, Hamburg, Germany

**Keywords:** Spinal astrocytoma, Next-generation sequencing, Prognostic factor, Molecular profile

## Abstract

Primary spinal cord astrocytomas are rare, hence few data exist about the prognostic significance of molecular markers. Here we analyze a panel of molecular alterations in association with the clinical course. Histology and genome sequencing was performed in 26 spinal astrocytomas operated upon between 2000 and 2020. Next-generation DNA/RNA sequencing (NGS) and methylome analysis were performed to determine molecular alterations. Histology and NGS allowed the distinction of 5 tumor subgroups: glioblastoma IDH wildtype (GBM); diffuse midline glioma H3 K27M mutated (DMG-H3); high-grade astrocytoma with piloid features (HAP); diffuse astrocytoma IDH mutated (DA), diffuse leptomeningeal glioneural tumors (DGLN) and pilocytic astrocytoma (PA). Within all tumor entities GBM (median OS: 5.5 months), DMG-H3 (median OS: 13 months) and HAP (median OS: 8 months) showed a fatal prognosis. DMG-H3 tend to emerge in adolescence whereas GBM and HAP develop in the elderly. HAP are characterized by CDKN2A/B deletion and ATRX mutation. 50% of PA tumors carried a mutation in the PIK3CA gene which is seemingly associated with better outcome (median OS: PIK3CA mutated 107.5 vs 45.5 months in wildtype PA). This exploratory molecular profiling of spinal cord astrocytomas allows to identify distinct subgroups by combining molecular markers and histomorphology. DMG-H3 tend to develop in adolescence with a similar dismal prognosis like GBM and HAP in the elderly. We here describe spinal HAP with a distinct molecular profile for the first time.

**Supplementary Information:**

The online version contains supplementary material available at 10.1186/s40478-021-01222-6.

## Introduction

Since the revision of the WHO classification of central nervous tumors in 2016, molecular alterations are part of the integrated diagnosis of glial tumors [22]. The overwhelming impact of molecular markers has further been revealed as summarized by the latest cIMPACT-NOW update [21–23].

Astrocytic tumors of the spinal cord are rare, especially spinal glioblastoma which account for approximately 1.5% of all spinal cord tumors [40, 41]. The majority of spinal cord astrocytomas are located cervicothoracic and usually have a short clinical history [11, 15]. Initial action in the course of treatment consists of surgery to obtain tissue for histological analysis and molecular specification. The value of chemotherapy beyond radiation therapy has yet to be determined as there are no data from dedicated trials available.

Next-generation sequencing (NGS) in astrocytomas in clinical networks is thought to improve diagnostic accuracy, prognostication and discovery of potentially druggable targets.

A Lysine-27-to-methonine (K27M) mutation at one allele of histone H3 in histologically defined diffuse intrinsic pons gliomas (DIPG), midline gliomas (such as thalamic tumors) and spinal cord astrocytomas has been found to be associated with an aggressive clinical course. This introduced the novel entity ‘diffuse midline glioma, H3 K27-mutand’ which is represents approximately 80% of diffuse intrinsic pontine gliomas (DIPGs), 50% of thalamic gliomas, and 60% of spinal cord gliomas [10, 18, 22, 24]. Recently, it was suggested that the term diffuse midline glioma H3 K27M-mutant should be reserved for tumors that are diffuse (i.e., infiltrating), midline (e.g., thalamus, brainstem, spinal cord, etc.) gliomas with the H3 K27M mutation, and not for other tumors with this mutation, such as ependymomas or midline gangliogliomas [21]. Therefore, treatment decisions are more and more based on molecular profiles of the specific tumor entity, especially if these genetic alterations provide potential targets for therapeutic agents.

To date, there is a lack of information regarding the molecular signature of spinal cord astrocytomas defining potentially prognostic subgroups. To further stratify and characterize the molecular profile of spinal astrocytomas in relation to the clinical outcome we conducted a retrospective study using a next-generation sequencing and methylome analysis.

## Materials and methods

### Study design

In this retrospective analysis we included patients undergoing surgical resection of primary astrocytoma or glioblastoma of the spinal cord at our institution between 2000 and 2020. Tumors were classified according to the WHO classification of tumors of the central nervous system (2016). We explicitly excluded ependymomas by histological diagnosis, as these have been studied in detail before [44].

Demographic, surgical, and histologic parameters, adjuvant treatment strategies (radio- or chemotherapy use) and neurological outcome were retrieved from the medical records. Surgical treatment was divided into gross total resection (GTR) and subtotal resection (STR) determined on the basis of early postsurgical imaging. The primary endpoint was overall survival (OS).

The cohort was further divided into 5 subgroups, by their unique molecular and histomorphological profile consisting of: glioblastoma multiforme (GBM), diffuse-midline gliomas H3 mutated (DMG-H3), high-grade astrocytoma with piloid features (HAP), diffuse astrocytoma (DA), diffuse leptomeningeal glioneuronal tumor (DLGNT) and pilocytic astrocytoma (PA).

Surgical approach consisted of posterior hemi-laminectomy or laminectomy. Intraoperative neuro-monitoring, ultrasound and microscope were used in all cases. Follow-up was done subsequently in our outpatient department every 3 months with MRI scans.

Tumor progression was defined by either clinical deterioration related to the spinal cord function, new contrast-enhancement or > 25% volume increase of residual contrast enhancement.

### Neuropathological evaluation and microscopy

Routine neuropathological evaluation of formalin fixed spinal cord glioma samples including hematoxylin and eosin as well as reticulin fiber staining. In addition, immunostaining with antibodies was performed using Ki67 and Olig2.

All histological samples were reviewed by an experienced neuropathologist (MMD) and re-classified based on the 2016 WHO classification of brain tumors [22]. The final diagnoses reported here are based on the combination of histology, genetics and (where available) methylome classifier results including the copy-number profile, which is generated as part of the methylome analysis. IDH-wildtype gliomas lacking necrosis or endothelial proliferation, but with molecular features of glioblastoma are reported here as glioblastoma, IDH wildtype as suggested in the cIMPACT-NOW update 6 [23].

Images were taken on Olympus BX51 microscope, equipped with an Olympus SC30 digital camera, or on a Zeiss Axioscan.Z1, using 20 × objectives. Microscope images shown were digitally adjusted for white balance, exposure, contrast and color saturation.

### DNA and RNA isolation

DNA and, for cases since 2018, RNA, were extracted from micro-dissected FFPE tissue using an automated Maxwell system for DNA (Promega, Fitchburg, Massachusetts, USA) or Allprep DNA/RNA Mini Kit (Quiagen, Hilden, Germany) according to the manufacturer’s instructions. Nucleic acid concentrations were determined on a on a Quantus Fluorometer (Promega, Walldorf, Germany) according to the manufacturer’s instructions.

### DNA methylation profiling

DNA methylation profiling was performed on cases from which sufficient DNA could be extracted from the tissue. Methylation profiling was done using 100–250 ng DNA on an Illumina Infinium MethylationEPIC BeadChip array (Illumina, San Diego, CA, USA), using the plate reader feature of an Illumina NextSeq 550. The protocols were according to the manufacturer’s instructions. Raw methylation data were analyzed using the DNA methylation-based brain tumor classifier (v11b4), which also provides data on copy-number alterations and MGMT promoter methylation [9]. T-stochastic neighborhood embedding (t-SNE) was calculated in R. Presumably because of DNA degradation, many older cases tended not to match definitively (score >  = 0.9), but a high score (> = 0.5) with compatible histology was deemed sufficient for a diagnosis. The methylation classes and associated scores are reported in detail in the Additional file 2: Table S1.

### DNA and RNA sequencing

On initial cases DNA panel sequencing was performed using an Illumina custom amplicon panel which targeted the coding DNA sequence or mutational hotspots of 34 brain tumor associated genes as well as selected regions on chromosomes 1 and 19 for copy-number analysis (Additional file 2: Table S1.). Library preparation was performed according to the manufacturer’s instructions using 50–100 ng DNA. Sequencing was performed on an Illumina MiniSeq.

From early 2019 on, we switched to the commercially available Illumina Trusight Oncology 500 (TSO500) panel for all clinical applications, which is a combined DNA and RNA panel. Thus, all recent cases, as well older cases which had become clinically relevant since that time, were sequenced using the TSO500 panel, which covers the coding DNA sequence of 522 cancer-associated genes, the TERT promoter, and the RNA sequence of 55 genes to detect fusions and splice variants. Library preparation was performed according to the manufacturer’s instructions using 30–150 ng DNA and 50–85 ng RNA. Sequencing was performed on an Illumina NextSeq 550.

Raw sequencing data were de-multiplexed and aligned to the human genome (GRCh37/hg19) using Illumina software, either on the MiniSeq for the custom panel or a dedicated Unix Server for the TSO500 panel, yielding variant calls, DNA amplifications, as well as fusions and splice variants. Variant calls were further evaluated using custom scripts written in Igor Pro (Wavemetrics, Lake Oswego, OR, USA) which retrieved variant effect prediction data from the ENSEMBL server using a REST API. Variants were subsequently filtered based on variant allele frequency (≥ 10%) and minor allele frequency in the population (≤ 1%). Variants annotated as “benign” or “likely benign” in ClinVar were rejected. Variants without a ClinVar entry were filtered both on SIFT and PolyPhen predictions, with variants being rejected which had both SIFT “tolerated” and PolyPhen “benign” as entries.

### Statistical analysis

SPSS for Windows (SPSS, Version 26.0, Chicago, IL) was used for statistical calculations. OS and PFS were analyzed with the Kaplan–Meier-method. The distribution of patient- and tumor-related variables was analyzed by chi-squared statistics (for categorical variables) and Kruskal–Wallis-Test (for continuously scaled variables). The median was used as threshold for dichotomization of parameters. For univariate prognostic analyses, all parameters were evaluated using Cox-regression. A two-tailed *p*-value < 0.05 was considered significant.

## Results

### Patient characteristics

In total 26 patients (19 male/7 female) with newly diagnosed astrocytomas were included with sufficient amount and quality of tissue for molecular analyses. The integrated diagnoses, based on histology, sequencing and DNA-methylation patterns were pilocytic astrocytoma (n = 8 patients), glioblastoma, IDH wildtype (n = 6 patients), diffuse midline glioma, H3 K27M mutated (n = 5 patients), high-grade astrocytoma with piloid features (n = 4 patients), diffuse astrocytoma, IDH mutated (n = 2 patients) and diffuse leptomeningeal glioneuronal tumor (n = 1 patients). None of these patients were known to have familial tumor predisposition syndromes based on anamnesis. Their clinical characteristics are summarized in Table 1. The length of total follow-up was 19 months.

**Table 1:** Patient characteristics and histomorphology

	GBM	DMG-H3	HAP	DA	DLGN	PA
Total	6	5	4	2	1	8
Age (years)	51.1 (11.6–73.8)	18.3 (9.3–53.3)	70.3 (44–72.9)	44.3 (33.5–55.1)	21.5	14.9 (2.2–48.9)
Gender (male/female)	5/1	4/2	4/0	1/0	1/1	4/3
Localization (cervical/thoracic/lumbar)	4/2/0	3/2/0	1/3/0	2/0/0	0/2/0	3/4/0
EOR (GTR)	0	0	0	0	0	2
Adjuvant RT	5	5	4	2	0	3
Adjuvant CT	5	4	4	1	0	0
Ki67 (median)	0.25	0.2	0.1	0.02	0.3	0.1
Necrosis	1	0	0	0	0	0
Endothelial Proliferation	3	3	0	0	0	0
Mitosis count (median)	8	2.5	1	0	0	0
OS (months)	5.5 (1–21)	13 (4–33)	8 (6–30)	72 (8–136)	141	80 (2–132)
Death	3	4	3	1	0	1

The median age at diagnosis was 14.9 years for PA, 18.3 years for DMG-H3, 21.5 years for DGLN, 44.3 years for DA, 51.1 years for GBM and 70.3 years for HAP patients, respectively. We correlated the genetic data of all tumors with available clinical parameters. Age varied considerably with a significantly younger patient population in PA, DLGNT and DMG-H3 tumors. The thoracic (n = 13) and cervical (n = 13) region were affected equally often. When further stratified by tumor grade, patients with higher WHO grade tumors were more likely to receive adjuvant RT than patients with pilocytic astrocytomas.

## Molecular characteristics

**Fig. 1:**
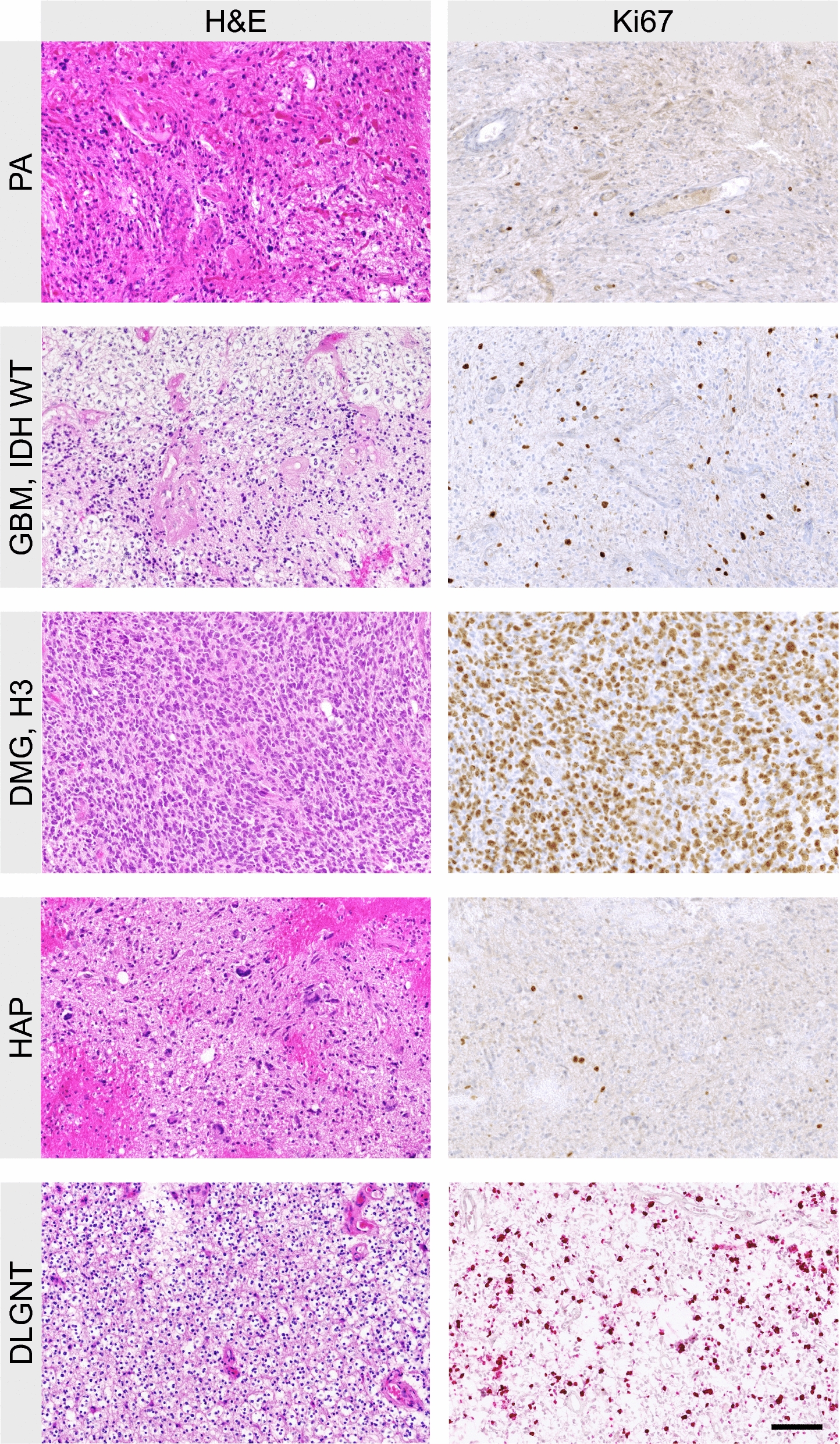
Histological features of spinal astrocytomas, hematoxylin and eosin stainings (H&E; left column) and Ki67 immunohistochemistry (right column). PA: pilocytic astrocytoma; GBM: glioblastoma IDH-wild-type; DMG-H3: diffuse midline glioma, H3 K27M mutated; HAP: high-grade astrocytoma with piloid features; Astro, IDH: diffuse astrocytoma IDH 1/2 mutated; DLGNT: diffuse leptomeningeal glioneuronal tumor Scale bar, 100 µm

**Fig. 2:**
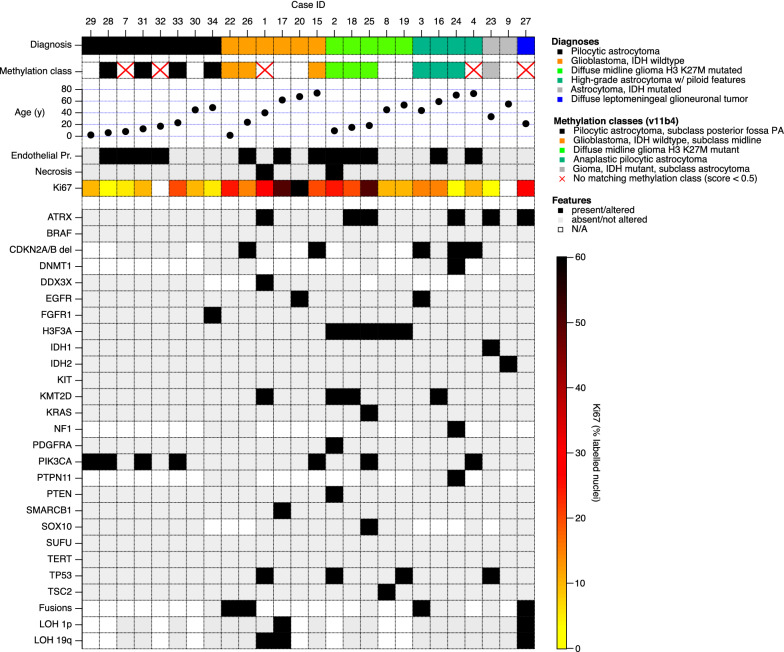
Characteristics of spinal astrocytomas. Diagnoses are based on the integration of histological assessment, molecular genetics and, where available, methylation classifier results. The presence of endothelial proliferation and necrosis was assessed by H&E histology, the Ki67 labelling index by immunohistochemistry. LOH 1p/19q, loss of heterozygosity at 1p/19q

**Fig. 3:**
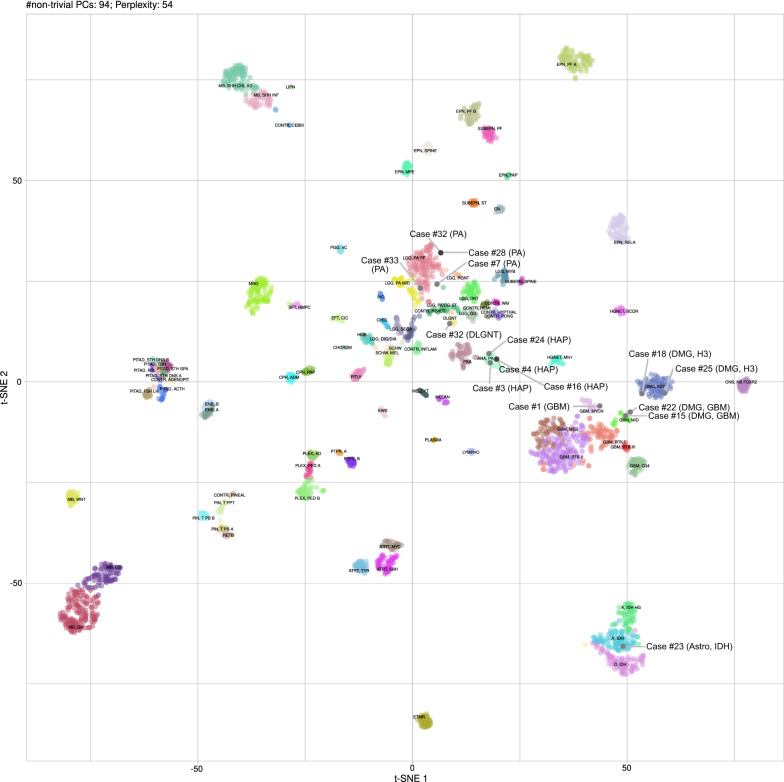
t-SNE clustering of analyzed cases against the DKFZ reference dataset [9]. Reference cases are colored according to the respective methylation classes. A: pilocytic astrocytoma; GBM: glioblastoma IDH-wild-type; DMG-H3: diffuse midline glioma, H3 K27M mutated; HAP: high-grade astrocytoma with piloid features; Astro, IDH: diffuse astrocytoma IDH 1/2 mutated; DLGNT: diffuse leptomeningeal glioneuronal tumor. Abbreviations of reference classes: A, IDH: astrocytoma, IDH mutant; A, IDH HG: astrocytoma, IDH mutant, high-grade; ANA, PA: high-grade astrocytoma with piloid features; ATRT, MYC: atypical teratoid/rhabdoid tumor, MYC-associated; ATRT, SHH: atypical teratoid/rhabdoid tumor, sonic hedgehog/Notch-associated; ATRT, TYR: atypical teratoid/rhabdoid tumor, tyrosinase-associated; CHGL: chordoid glioma; CHORDM: chordoma; CN: central neurocytoma; CNS, NB FOXR2: CNS neurolastoma, FOXR2-activated; CONTR, ADENOPIT: control tissue, adenohypophysis; CONTR, CEBM: control tissue, cerebellar hemisphere; CONTR, HEMI: control tissue, cerebral hemispheric cortex; CONTR, HYPTHAL: control tissue, hypothalamus; CONTR, INFLAM: control tissue, inflammatory tumor microenvironment; CONTR, PINEAL: control tissue, pineal gland; CONTR, PONS: control tissue, pons; CONTR, REACT: control tissue, reactive tumor microenvironment; CONTR, WM: control tissue, white matter; CPH, ADM: adamantinomatous craniopharyngeoma; CPH, PAP: papillary craniopharyngeoma; DLGNT: diffuse leptomeningeal glioneuronal tumor; DMG, K27: diffuse midline glioma, H3 K27M mutant; EFT, CIC: CNS Ewing Sarcoma Family Tumor with CIC alteration; ENB, A: esthesioneuroblastoma, subclass A; ENB, B: esthesioneuroblastoma, subclass B; EPN, MPE: myxopapillary ependymoma; EPN, PF A: ependymoma, posterior fossa A; EPN, PF B: ependymoma, posterior fossa B; EPN, RELA: ependymoma, RELA fusion positive; EPN, SPINE: ependymoma, spinal; EPN, YAP: ependymoma, YAP fusion positive; ETMR: embryonal tumor with multilayered rosettes; EWS: Ewing sarcoma; GBM, G34: glioblastoma, IDH wildt-type, H3-3 G34 mutant; GBM, MES: glioblastoma, IDH wild-type, mesenchymal; GBM, MID: glioblastoma, IDH wild-type, midline; GBM, MYCN: glioblastoma, IDH wild-type, MYCN-associated; GBM, RTK I: glioblastoma, IDH wild-type, receptor tyrosine kinase I; GBM, RTK II: glioblastoma, IDH wild-type, receptor tyrosine kinase II; GBM, RTK III: glioblastoma, IDH wild-type, receptor tyrosine kinase III; HGNET, BCOR: CNS high grade neuroepithelial tumor with BCOR alteration; HGNET, MN1: CNS high grade neuroepithelial tumor with MN1 alteration; HMB: hemangioblastoma; IHG: infantile hemispheric glioma; LGG, DNT: low-grade glioma, dysembryoplastic neuroepithelial tumor; LGG, GG: low-grade glioma, ganglioglioma; LGG, MYB: low-grade glioma, MYB/MYBL1 altered; LGG, PA MID: low-grade glioma, midline pilocytic astrocytoma; LGG, PA PF: low-grade glioma, posterior fossa pilocytic astrocytoma; LGG, PA/GG ST: low-grade glioma, hemispheric pilocytic astrocytoma and ganglioglioma; LGG, RGNT: low-grade glioma, rosette-forming glioneuronal tumor; LGG, SEGA: low-grade glioma, subependymal giant-cell astrocytoma; LIPN: cerebellar liponeurocytoma; LYMPHO: lymphoma; MB, G3: medulloblastoma, group 3; MB, G4: medulloblastoma, group 4; MB, SHH CHL ADL: medulloblastoma, sonic hedgehog activated A (children/adult); MB, SHH INF: medulloblastoma, sonic hedgehog activated B (infant); MB, WNT: medulloblastoma, WNT activated; MELAN: malignant melanoma; MELCYT: melanocytoma; MNG: meningioma; O, IDH: oligodendroglioma, IDH-mutant; PGG, nC: paraganglioma, spinal non-CIMP; PIN, T PB A: pineoblastoma group A / intracranial retinoblastoma; PIN, T PB B: pineoblastoma group B; PIN, T PPT: pineal parenchymal tumor; PITAD, ACTH: pituitary adenoma, ACTH; PITAD, FSH LH: pituitary adenoma, FSH/LH; PITAD, PRL: pituitary adenoma, prolactin; PITAD, STH DNS A: pituitary adenoma, STH densely granulated, group A; PITAD, STH DNS B: pituitary adenoma, STH densely granulated, group B; PITAD, STH SPA: pituitary adenoma, STH sparsely granulated; PITAD, TSH: pituitary adenoma, TSH; PITUI: pituicytoma / granular cell tumor / spindle cell oncocytoma; PLASMA: plasmacytoma; PLEX, AD: plexus tumor, subclass adult; PLEX, PED A: plexus tumor, subclass pediatric A; PLEX, PED B: plexus tumor, subclass pediatric B; PTPR, A: papillary tumor of the pineal region, group A; PTPR, B: papillary tumor of the pineal region, group B; PXA: pleomorphic xanthoastrocytoma; RETB: retinoblastoma; SCHW: schwannoma; SCHW, MEL: melanotic schwannoma; SFT, HMPC: solitary fibrous tumor / hemangiopericytoma; SUBEPN, PF: subependymoma, posterior fossa; SUBEPN, SPINE: subependymoma, spinal; SUBEPN, ST: subependymoma, supratentorial;

Tumors were classified based on histologic and genetic alterations, as well as the results of methylome analysis, where available. Samples of histological properties are shown in Fig. 1, while Fig. 2 summarizes the overall genomic alterations of all patients, with details listed in the Supplementary Table. Results of t-SNE clustering of cases on which methylome analysis was performed are shown in Fig. 3.

IDH-wildtype glioblastomas were histologically and molecularly heterogeneous. Fusion genes were detected in the two youngest patients: Case #22, a 1.6 years old boy, had a TPM3-NTRK1 fusion, and case #26, a 24 years old male, had a FGFR1-PLEC fusion as well as a PDGFRA-FIP1L1 fusion. Both these tumors, however, showed a match to the methylation class “glioblastoma, IDH wildtype, subclass midline” with high calibrated scores (0.99 and 0.97, respectively), confirming the diagnosis of glioblastoma, IDH wildtype.

None of the spinal glioblastomas had a TERT promoter mutation, while typical copy-number alterations such as CDKN2A/B deletion or CDK4 amplification were observed in some tumors. Only one single glioblastoma had an ATRX mutation with concomitant loss of nuclear protein expression. Although TP53 mutations are reported in up to 37.5% of intracranial primary GBM, only one patient carried a TP53 mutations [8]. Interestingly, beside TP53 mutation, #1 carried multiple mutations (ATRX, DDX3X and KMT2D) and signs of necrosis, hypothesizing a more aggressive phenotype. Three tumors showed methylation of the MGMT promoter region.

Diffuse H3 mutated midline gliomas all harbored a lysine to methionine mutation on codon 28 of the histone gene H3-3A, which is commonly referred to as “K27M” [20]. Additional mutations were heterogeneous, with two tumors each showing frameshift or stop mutations in ATRX and KMT2D. Overall mutational load is higher in DMG-H3 tumors compared to any other malignant tumor subtype (GBM, APP) with an average of 3 mutations per tumor (GBM: 2, HAP: 2.5). Histologically, three tumors showed features of malignancy while the remaining two did not. The latter showed significantly lower number of mutations (median n = 2) (Spearman-Rho: r = 0.973, p = 0.005) and were older (median age = 49.3 years), than their more aggressive counterparts (median n = 5 mutations, median age = 15.1 years).

High-grade astrocytomas with piloid features occurred in elderly patients compared to PA (median age 70.3 yrs vs 14.9 yrs, *p* = 0.016). Some of these tumors showed endothelial proliferation and appeared histologically as malignant gliomas (Additional file 1: Figure S1.). Most of these tumors showed a homozygous CDKN2A/B deletion. ATRX mutation was confirmed in one patient. MGMT promoter region was methylated in all HAP. Methylation analysis led to a reclassification into a distinct group of these four cases.

IDH mutated astrocytomas were rare, with one IDH1 and one IDH2 mutated tumor in our cohort. While the former had an ATRX mutation and TP53 mutation, like its supratentorial counterparts, the latter lacked further mutations. However, infratentorial IDH mutated astrocytomas were recently described as a separate subgroup, often lacking the typical combination of ATRX and TP53 mutations [3].

Pilocytic astrocytomas showed typical histology, often with prominent Rosenthal fibers and endothelial alterations but low Ki67 labelling indices. Within PA, of all analyzed genes, PIK3CA gene was most frequently altered in 50% of which E545A being the most common. Tumors with PIK3CA alterations did not differ in age (*p* = 0.149) or localization (*p* = 0.102) compared to PIK3CA wildtype. PIK3CA mutation was associated with longer OS compared to PIK3CA wildtype (median OS: 107.5 vs 45.5 months). One pilocytic astrocytoma which was wildtype in PIK3CA harbored a FGFR1 N546K mutation. Unfortunately, no RNA data was available for any of the pilocytic astrocytomas, so we were not able to assess BRAF or FGFR fusion genes.

One single tumor was classified as diffuse leptomeningeal glioneuronal tumor, with oligodrendroglia-like morphology, loss of ATRX expression, KIAA1549-BRAF fusion and 1p/19q co-deletion. Clinically, this tumor was characterized by multiple relapses over a course of almost 17 years.

## Treatment

Two patients harboring a PA received gross total resection and 24 patients underwent subtotal resection. 19 patients received adjuvant radiation therapy immediately following diagnosis (5 GBM, 5 DMG-H3, 4 HAP, 2 DA and 3 PA). Chemotherapy was administered in 5 patients with GBM, 4 patients with DMG-H3, 4 patients with HAP and one patient with DA.

## Patient’s outcome: univariate

**Fig. 4:**
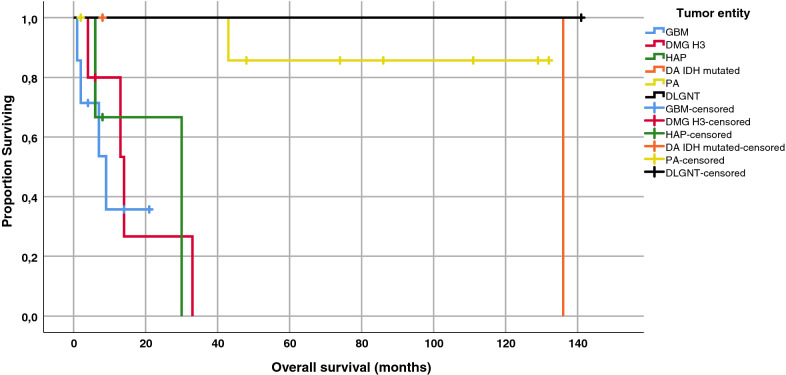
Kaplan–Meier analysis of overall survival stratified by subgroup (GBM, DMG-H3, HAP, DA, DLGNT and PA)

12/26 patients died: 3/6 GBM, 4/5 DMG-H3 3/4 HAP, 1/2 DA and 1/8 PA—the DLGNT tumor patient was alive at the time of last follow-up. The clinical prognosis was significantly associated with tumor subtype (p = 0.002), H3 K27M-mutation (P = 0.03), male sex (p = 0.05) and age (p = 0.002), in univariate Cox regression analysis (Table 2). Tumors harboring a mutation in the H3-3A-gene presented with a significantly more aggressive clinical course, like GBM IDH wild type or HAP. Survival was shortest in GBM, DMG-H3 or HAP with no significant difference in Kaplan–Meier analyses. ATRX status (mutation versus ATRX wild-type) was not associated with survival, neither was presence or absence of necroses (Table 1). The Kaplan–Meier estimates of OS are shown in Fig. 4. As PIK3CA mutations were found in 50% of PA, we conducted a subgroup analysis which revealed an association with longer OS in our cohort (median OS: PIK3CA mutated 107.5 vs 45.5 months in wildtype PA). Due to individualized treatment concepts, including microsurgical resection, chemotherapy and radiation no conclusion can currently be drawn as to the effectiveness of particular therapeutic strategies.

**Table 2:** Univariate analysis

	Univariate analysis		
	*p*-value	HR	95% CI for HR
Age (continuously)	**0.002**	**1.063**	**1.022–1.105**
Female Gender (vs. male)	**0.05**	**0.127**	**0.016–0.998**
Localization (cervical vs. thoracic)	0.228	0.509	0.170–1.526
Mutation H3F3A (yes vs.no)	**0.03**	**4.164**	**1.038–15.801**
ATRX loss (yes vs. no)	0.085	0.119	0.011–1.337
Necrosis (yes vs. no)	0.650	0.045	0.136–9.092
Endothelial Proliferation (yes vs. no)	0.222	2.108	0.637–6.977
Mitosis (continuously)	0.059	1.653	0.981–2.784
Ki-67 (continuously)	0.057	10.428	0.75–43.398
Tumor Subtype	**0.002**	**0.42**	**0.244–0.724**
Adjuvant Treatment (yes vs no)	0.154	3.063	0.657–14.289

## Discussion

Since the revision of the WHO classification of central nervous system tumors in 2016 tumors are classified by an integrated diagnosis combining histologic and molecular features [22]. Astrocytomas of the spinal cord are infrequent and up-to-date knowledge of their respective tumor biology and molecular alterations is sparse. Thus, a consensus on the treatment of spinal cord astrocytomas according to the biology of these tumors has yet to be determined [11]. Due to the infiltrating nature of these tumors safe gross total resection is rarely feasible, especially within the high-grade counterparts [16, 25]. Current treatment strategies for diffuse midline gliomas, as recommended by the EANO guidelines, consist of radiotherapy alone or TMZ plus radiotherapy followed by TMZ as first line therapy [37]. Our study focused on the clinical, histological and molecular characterization of spinal cord gliomas. To gain more insight into potentially clinically relevant molecular alterations we additionally analyzed a large set of molecular markers in all tumors.

Mutations in genes affecting the histone code and alteration of histone methylation status have been detected in several cancer entities. Interestingly, mutations within a histone gene were initially discovered in gliomas [19, 39]. Mutation in the K27M position of the histone H3-3A gene leads to a block of methylation on the histone H3 tail inhibiting the cascade responsible for glial differentiation [12]. Other entities such as ependymomas, subependymomas and glioneuronal tumors may rarely harbor H3K27M mutations, however their prognostic value in these tumors is less clear [27, 28, 32, 42].

We found 5/26 (19.2%) H3-3A K27M positive cases. Ishi et al. found 18.9% of H3-3A K27M mutations in adult patients while Yi et al. reported a frequency of 80% (20 out of 25 patients) in a similar sized collective compared to our study [17, 43].

In accordance with previous reports the H3-3A K27M mutation is associated with a fatal prognosis mimicking the clinical course of grade IV tumors like glioblastoma multiforme WHO grade IV. However, most of the published studies included intracranial tumor manifestations resulting in a significant underrepresentation of spinal cord localization. The mutated tumors were associated with worse prognosis in comparison to H3-3A wild-type tumors regardless of tumor grade or location [6, 7, 13]. Our study confirmed these results for DMG-H3 spinal gliomas without any prognostic distinction regarding proliferation or histological grading within this subgroup. However, our sample size of H3-3A K27M mutated tumors did not allow further statistical analysis. Chai et al. and others found a longer survival for H3-3A K27M-mutanted gliomas with less anaplastic features compared to H3-3A K27M-mutant WHO grade IV or H3-3A K27M-wild-type WHO grade IV gliomas, suggesting a prognostic distinction of histomorphological appearance [10, 45]. In contrast, Yi et al. reported a more favorable prognosis of H3-3A K27M mutant tumors compared to H3-3A K27M-wild-type without addressing the histomorphological appearance within the H3-3A K27M-mutant cohort [43].

IDH-wildtype glioblastoma in our cohort were heterogeneous, both clinically and molecularly, suggesting that several distinct molecular entities are currently subsumed in this diagnosis. For instance, the tumor of youngest patient (case #22) had a NTRK1 fusion gene, which is typical for a subgroup of infant hemispheric gliomas with receptor-tyrosine kinase activation, yet the current methylome classifier (v11b4) produced a high calibrated score (0.99) for the methylation class “glioblastoma, IDH wildtype, subclass midline” [14]. Similarly, case #26, a malignant glioma with FGFR1 fusion and deletions of CDKN2A/B and TP53 in a young patient, matched to this group with a calibrated score of 0.97. As a match to a glioblastoma methylation class is considered to be a molecular feature of glioblastoma and due to the current lack of a better suited tumor class, we classified these tumors as glioblastoma [5]. Nevertheless, their respective clinical and molecular features may warrant different diagnoses in the future.

None of the glioblastomas in our cohort had a TERT promoter mutation, which is common in the supratentorial counterparts. However, another recent study on spinal cord gliomas described three cases harboring TERT promoter mutations, suggesting that these do occur in spinal glioblastoma, though probably at a lower frequency than in non-spinal glioblastoma [2].

In our study patients with either DMG-H3 (median OS: 13 months), GBM (median OS: 5.5 months) or HAP (median OS: 8 months) had worse survival rates compared to DA (median OS: 72 months) or PA (median OS: 141 months). Patients with DMG-H3 were predominantly younger in accordance with previous reports [1, 26, 34]. Thus, we assume an analogy of the grim prognosis in spinal wild-type GBM in elderly and DMG-H3 in young age. In a study by Perwein et al. dealing with pediatric spinal astrocytomas no patient showed a K27M alteration of the H3-3A gene, however this result was solely based on immunohistochemistry [29]. Interestingly, we found high proliferating activity within DMG-H3 tumors to be associated with a high mutational burden. High mutational burden has been described to be linked with worse outcome in supratentorial gliomas [36]. Larger cohorts have to be analyzed to address this question in spinal gliomas as well.

Within the DA cohort, which had a longer median OS compared to HAP, DMG-H3 and GBM, we found two tumors with a IDH 1 or 2 mutation (n = 1 each). Since only one similar case has been reported up to now, the prognostic value of IDH 1/2 mutations has yet to be determined in larger, potentially collaborative studies [35].

HAP have been defined as a distinct, molecular entity recently [30], with only few cases reported in the spinal cord [4]. A definitive diagnosis of these tumors currently relies on methylation analysis [23]. We found several spinal HAP, which had an unfavorable clinical course with a short median OS of 8 months, which is considerably shorter than reported previously for tumors primarily stemming from locations outside the spinal cord [4, 30].

For the first time we here present HAP being located in the spinal cord, these patients had a short median OS of 8 months. These tumors with histomorphological characteristics of PA, but with increased mitotic index and other high-grade features have so far been described for intracranial gliomas (Additional file 1: Figure S1.). They harbor alterations in the MAPK pathway as well as alterations of CDKN2A/B and ATRX and display a fatal clinical course [30]. In line with other studies, we also found a higher median age of patients with HAP compared to PA [30, 31]. We were also able to confirm in HAP alterations in CDKN2A/B in three tumors and an ATRX alteration in one specimen. The MGMT promoter region was methylated in all cases, thus significantly higher in comparison to their intracranial counterparts (45%) [30].

In PA, we could detect a mutation of the PIK3CA gene in 50% of our patients, which are rarely reported in pilocytic astrocytomas outside the spinal cord. Among low-grade CNS tumors, PIK3CA mutations have been described in rosette-forming glioneuronal tumors although they also occur in glioblastoma [33, 38]. Interestingly, in our study PIK3CA mutation seems to be associated with longer OS, which may be caused by its association with pilocytic astrocytoma, which in turn had the most favorable overall outcome. This preliminary signal derived from a small cohort needs to be validated in a larger dataset.

This study is limited by its retrospective study design and the heterogeneity of postsurgical treatment regimes. Moreover, comparison between subgroups is hampered by small patient numbers. Nevertheless, the analysis of larger panels of molecular markers might provide additional knowledge of tumor biology and clinical behavior in spinal gliomas as has been found for the cerebral counterparts in the past.

## Conclusion

Spinal astrocytomas are comprised of histomorphological heterogeneous subgroups of tumors with distinct molecular alterations. DMG-H3 tumors with H3-3A mutations tend to develop in adolescence and have a similar dismal prognosis like GBM and HAP in the elderly. HAP within the spinal cord, harboring a specific molecular signature, is described here for the first time, having a similar dismal prognosis as their supratentorial counterparts. PA with a mutated PIK3CA gene seemingly have longer OS.

Analysis of molecular alterations in spinal astrocytomas might be of added value for diagnostic accuracy and clinical management. Future collaborative clinical trials in larger cohorts are needed.

## Supplementary Information

**Additional file 1.** Characteristics of spinal high-grade astrocytomas with piloid features. Micropgraphs of H&E-stained sections of cases #3, #4, #16 and #23 are shown alongside Manhattan plots showing their respective copy-number profiles. Cases #3, #4, and #23 had deletions of the CDKN2A/B genes, highlighted by blue arrows. Scale bar, 100 µm

**Additional file 2.** Detailed results of histological assessment and molecular analysis.

## Data Availability

The dataset supporting the conclusions of this article is included within the article as supplementary material. Other data is available from the corresponding author on reasonable request.
