# Supplementary figures and images for: Molecular diagnostics helps to identify distinct subgroups of spinal astrocytomas
Source: Acta Neuropathol Commun. 2021 Jun 30;9:119. doi: 10.1186/s40478-021-01222-6 (PMC8244211; doi:10.1186/s40478-021-01222-6)

Case #3

H&E

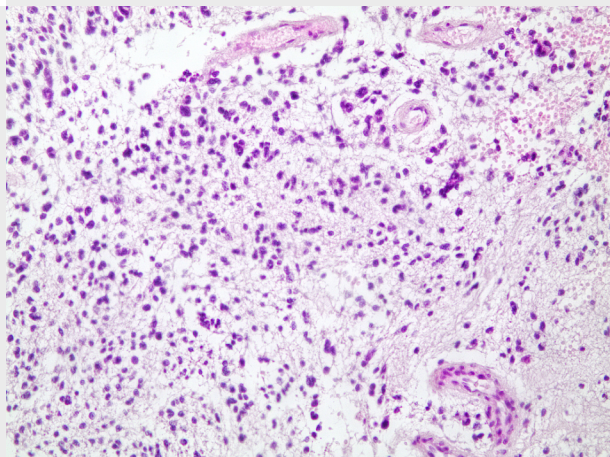

CNV plot

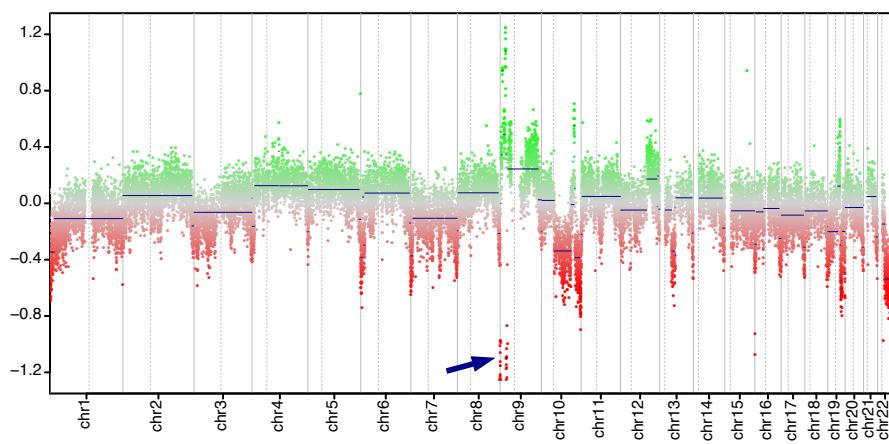

Case #4

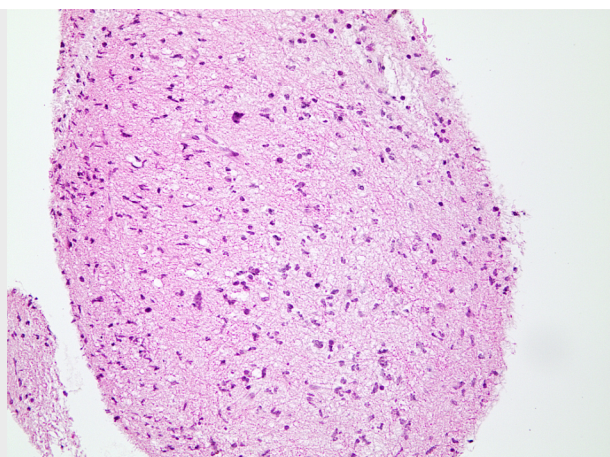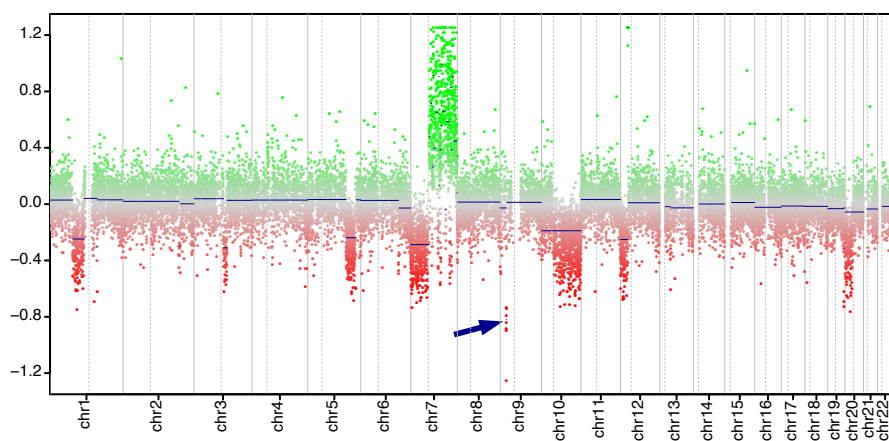

Case #16

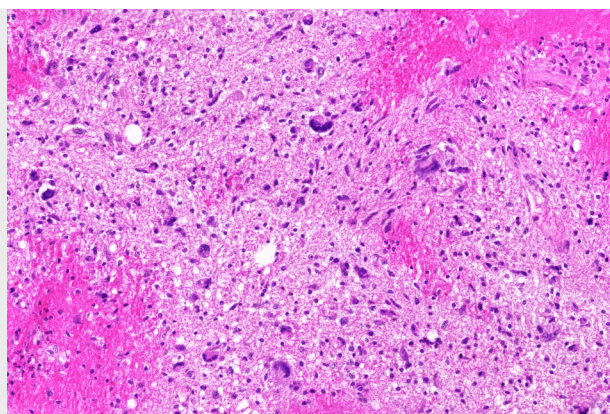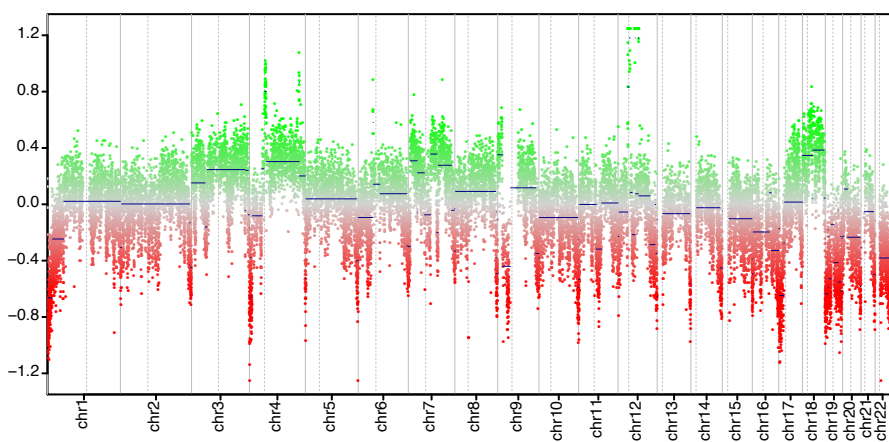

Case #24

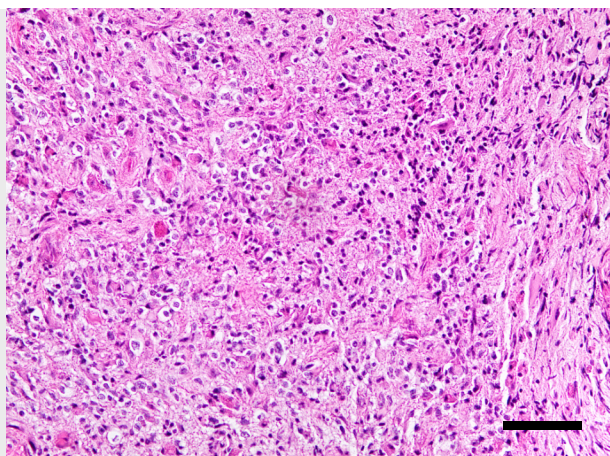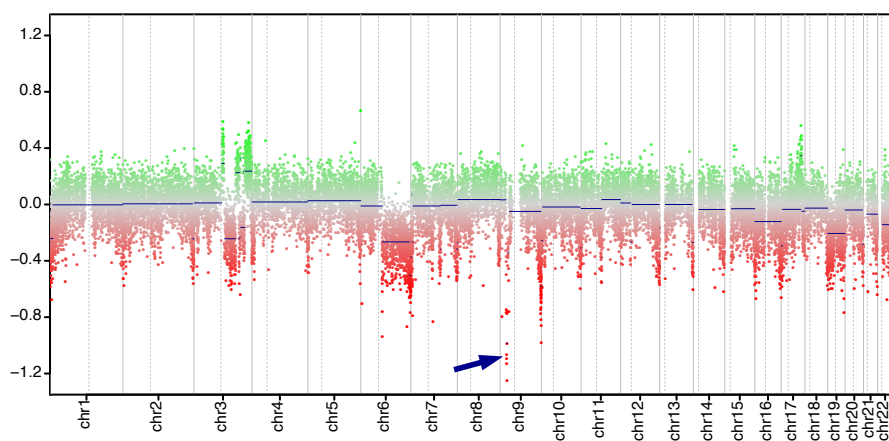

Supplement: Supplementary file 1 — Additional file 1. Characteristics of spinal high-grade astrocytomas with piloid features. Micropgraphs of H&E-stained sections of cases #3, #4, #16 and #23 are shown alongside Manhattan plots showing their respective copy-number profiles. Cases #3, #4, and #23 had deletions of the CDKN2A/B genes, highlighted by blue arrows. Scale bar, 100 µm [file 40478_2021_1222_MOESM1_ESM.pdf]
